# Association between behavioral patterns and depression symptoms: dyadic interaction between couples

**DOI:** 10.3389/fpsyt.2023.1242611

**Published:** 2023-11-16

**Authors:** Qianhui Yang, Xin Gao, Ying Tang, Hong Gan, Baoling Wang, Mengdie Li, Guixia Pan, Shuangshuang Bao, Peng Zhu, Shanshan Shao, Fangbiao Tao

**Affiliations:** ^1^Department of Maternal, Child and Adolescent Health, School of Public Health, Anhui Medical University, Hefei, Anhui, China; ^2^MOE Key Laboratory of Population Health Across Life Cycle, Hefei, Anhui, China; ^3^NHC Key Laboratory of Study on Abnormal Gametes and Reproductive Tract, Hefei, Anhui, China; ^4^Anhui Provincial Key Laboratory of Population Health and Aristogenics, Anhui Medical University, Hefei, Anhui, China; ^5^Department of Epidemiology and Biostatistics, School of Public Health, Anhui Medical University, Hefei, Anhui, China

**Keywords:** depression symptom, childbearing age, dyadic correlation, health-related behavior, couple

## Abstract

**Background:**

Behavioral patterns are sometimes associated with depression symptoms; however, few studies have considered the intra-couple effects. This study examined the effect of a spouses’ behavioral patterns on depression symptoms within themself and in their spouse.

**Methods:**

A total of 61,118 childbearing age participants (30,559 husband-wife dyads) were surveyed. The depression symptoms were assessed using the nine-item Patient Health Questionnaire (PHQ-9). The behavioral patterns were identified by the latent class analysis. The effects of behavioral patterns on the couple’s own depression symptoms (actor effect) and their partner’s depression symptoms (partner effect) were analyzed using the Actor-Partner Interdependence Model (APIM).

**Results:**

Three behavioral patterns were identified: low-risk group, moderate-risk group, and high-risk group. The high risk of these behavior patterns would be associated with a higher score on the PHQ-9; for both husbands and wives, their behavioral patterns were positively associated with PHQ-9 scores (β_husband_ = 0.53, *P* < 0.01; β_wife _= 0.58, *P* < 0.01). Wives’ behavioral patterns were also positively associated with their husbands’ PHQ-9 scores (β = 0.14, *P* < 0.01), but husbands’ behavioral patterns were not associated with their wives’ PHQ-9 scores.

**Conclusions:**

Wives’ depression symptoms were affected only by their own behavioral patterns, whereas husbands’ depression symptoms were influenced by both their own and their spouses’ behavioral patterns.

## 1. Introduction

Depression is a common mental illness that characterized by persistent low mood, diminished interest, and loss of pleasure (1). In China, the lifetime prevalence rate of depressive disorders had reached 6.9%, according to a recent large-scale survey (2). Depression in couples often affect marital quality, leading to increased divorce rates and potentially affecting the health of offspring (3–5). A meta-analysis evaluating five family studies showed a two- to three-fold increase in depression risk in the first-degree offspring of patients with depression (6). Moreover, the probability of depression is more than five times higher in a population with a depressed spouse than in those without a depressed spouse (7). Given the burden of depression and the correlation between couples, it is essential to understand the related modifiable risk factors (e.g., health-related behaviors) to design comprehensive and targeted interventions.

Recent data show that health-related behaviors—including drinking (8), smoking (9), dietary factors (10), physical inactivity (11), and sleep (12)—were associated with depressive symptoms. Additionally, these behaviors are codependent and should be considered simultaneously (13, 14). A cluster analysis study found that, compared with the relatively high-risk class, physically active and nonsmoking-and-nondrinking classes had lower probabilities of depression (15). However, most existing studies investigated depression and health-related behaviors among married people on individual-level determinants, rather than the couple dyad.

Family system theory states that family members are intensely emotionally connected, and that individuals interact with one another in a family (16–18). Couples influence each other and share various environmental factors and experiences. Many investigative studies have found that one partner’s life satisfaction (16), marital satisfaction (17), or physical activity (11) was associated with her/his spouse’s depression, while Donarelli et al. (19) found that psychological counseling for couples was effective in improving their psychological wellbeing at the dyadic level. However, few studies have focused on the impact of health-related behaviors on depression symptoms at the dyadic level of couples. Given the intrinsically dyadic nature of both the behaviors and the depression symptoms, a dyadic approach should be applied when the study subjects were couples (20, 21). The Actor-Partner Interdependence Model (APIM) is a statistical approach that can be applied to analyze dyadic interactions (22). It estimates the effects of an individual’s attributes on both their own outcome variable (actor effect) and that of their partner (partner effect). The APIM was recently used to simultaneously examine intra- and interpersonal effects among couples, such as that of marital satisfaction on depression (23, 24).

**FIGURE 1:**
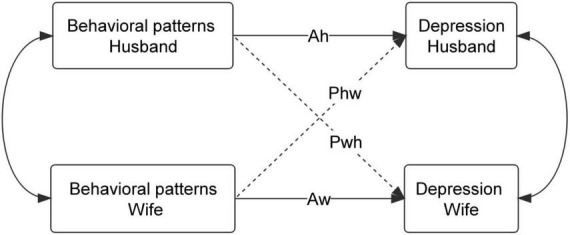
Actor–Partner Interdependence Model of behavioral patterns and depression. Ah: actor effect of husband’s behavioral patterns on his own depression; Aw: actor effect of wife’s behavioral patterns on her own depression; Pwh: partner effect of husband’s behavioral patterns on wife’s depression; Phw: partner effect of wife’s behavioral patterns on husband’s depression.

This multicenter cross-sectional study used a Latent Class Analysis (LCA) to identify participants’ behavioral patterns, including various health-related behaviors, and applied the APIM framework to determine actor and partner effects of behavioral patterns on depression symptoms among couples of childbearing age (Women aged 20–49 years and men aged 22–49 years). We hypothesized that (1) behavioral patterns between couples are correlated, as is depression symptoms between couples; and (2) behavioral patterns would be associated with the individual’s own depression symptoms, as well as their spouse’s depression symptoms, in husband-wife dyads (see Figure 1).

## 2. Materials and methods

### 2.1. Study population

The *Reproductive Health of Childbearing Couples—Anhui Cohort* (RHCC-AC) study is ongoing. RHCC-AC is a large pre-pregnancy cohort study based on reproductive couples. The study aims to identify the independent and combined effects of pre-pregnancy lifestyles and environmental exposure on infertility, adverse pregnancy outcomes, and offspring growth and development. More details are given in our previous literature (25). We recruited 33,687 couples (33,271 men, 33,354 women, and 32,938 husband-wife dyads) from 16 sites in Anhui Province from April 2019 to June 2021. Women aged 20–49 years and men aged 22–49 years were eligible. Participants completed a baseline questionnaire regarding demographics, health-related behaviors, reproductive history, and mental health. All questionnaires were completed under the supervision of an investigator. They completed follow-up questionnaires every 6 months for up to 24 months to ascertain their pregnancy status. Informed consent was obtained from all the participants. This study was approved by the Ethics Committee of Anhui Medical University (Number: 20189999).

We excluded 1,319 couples who were included in the pre-survey or where one of the couples did not complete the baseline survey. In addition, we excluded 1,124 remarried couples, as well as 267 couples with missing health-related behavior data, and 418 couples with missing Patient Health Questionnaire (PHQ-9) data. The final data set thus included 30,559 couples (90.7%). A flow diagram of the participants is shown in Supplementary Figure 1.

### 2.2. Measures

#### 2.2.1. Depression symptoms assessment

Depression symptoms was measured using the PHQ-9, a widely used and well-validated patient-rated assessment tool for depressive symptoms (26, 27). The PHQ-9 comprises nine items to respond to by reviewing the past 2 weeks. Each item is scored on a 4-point scale from 0 (not at all) to 3 (nearly every day). Total scores range from 0 to 27 points, with higher scores indicating more severe depressive symptoms. It has demonstrated considerable reliability and validity, and has been used in numerous studies involving couples of childbearing ages (28, 29). In this study, the alpha Cronbach of the PHQ-9 was 0.80.

#### 2.2.2. Demographic and health-related behaviors information

A self-administered questionnaire was used to collect the demographic and health-related behaviors information. Demographic information included living region, educational level, annual income, age, and BMI. Health-related behaviors included smoking, drinking, chronotype, sitting time, and dietary habits. The different behavioral patterns were determined through latent class analysis (LCA).

*Smoking* was assessed—and participants classified accordingly—by asking “Do you smoke?” (smoking at least one cigarette per day for 6 months or more).

*Drinking* was assessed by asking “Have you been drinking in the last 6 months?” Possible responses were (1) never, (2) less than once per week, and (3) every week.

*Chronotype* was assessed using the Munich Chronotype Questionnaire. Chronotype is an instrument useful for studying circadian biology in humans. It recorded behavior separately, on school (or work) and school-free (or work-free) days. The chronotype was calculated as the midpoint of sleep on school/work-free days, corrected for sleep debt accumulated on school/workdays (30). The chronotype was expressed as the local clock time and ranged from early (early midpoint of sleep) to late (late midpoint of sleep) chronotypes. Previous studies have found that a late chronotype has been linked to an increased risk for depression, anxiety, and substance abuse among adults (31). We dichotomized the study population into two groups: early chronotypes and late chronotypes.

*Sitting time* was assessed using the International Physical Activity Questionnaires. Participants were asked about the time spent sitting or reclining on a typical day, including at work, at home, getting to and from places, or with friends. We dichotomized the study population into two groups: those sitting for at least 8 h per day (sedentary behavior) and those sitting for less than 8 h per day (32).

*Dietary habits* included the frequency of daily diet and the consumption of takeaway food. The content of daily diet were collected using Food Frequency Questionnaires, including the intake of fruits and vegetables, legumes and nuts, cereals, animal-source foods (eggs, red meat, poultry, aquatic products, milk), sugar-sweetened beverages, and pickled/fired/barbecued foods. For frequency, participants were asked, “During the last months, how often did you eat the items listed below?” Participants were categorized as “daily intake and more” and “less than daily intake” according to their responses on the questionnaires and the Dietary Guidelines for Chinese Residents (2022) (29). Intake of sugar-sweetened beverages and pickled/fired/barbecued foods were dichotomized into “more than three times per week,” “less than three times per week” (33). Data on takeaway food and disposable cutlery consumption were collected using two questions. “How many times did you eat takeaway food in the last week?” “How many times have you used disposable cutlery in the past week?” Consumption was dichotomized into “more than three times per week,” “less than three times per week,” as used in a previous study (29, 33).

### 2.3. Statistical analysis

All analyses were performed using SPSS statistical software (v23.0) and MPLUS statistical software (v8.0). Descriptive statistics—such as means, standard deviations, frequencies, and percentages—were used to describe participants’ characteristics. Spearman’s correlation analyses were used to determine the correlation between behavioral patterns and depression, behavioral patterns between couples, and depression between couples.

#### 2.3.1. LCA analysis

Latent Class Analysis was used to identify behavioral patterns, a statistical technique for describing unobserved (i.e., latent) subgroups from patterns of observed variables. It considers profile membership as an unobserved categorical variable indicating the probability of an individual belonging to a certain profile (34). An LCA is suitable for dichotomous variables and also enables the identification of distinct configurations of heterogeneity within a sample. In our study, health-related behaviors were classified as dichotomous variables according to the presence or absence of risk. Specifically, the risk of all health-related behaviors variable was determined using the categories “No” or “Yes” for analysis. The assignments and frequency distribution of each indicator are presented in Supplementary Tables 1, 2. The best-fitting model was obtained using the Akaike information criterion (AIC), Bayesian information criterion (BIC), and entropy values. The smaller the AIC and BIC values, the simpler the model and the better the fit [Supplementary Table 3; (35)]. Entropy values close to 1.0 indicate a clear delineation of classes, with values > 0.8, generally indicating good classification. When more than one model fits well, the best and simplest model is retained, based on parsimony and interpretability.

#### 2.3.2. APIM analysis

Figure 1 depicts the APIM framework of a husband-wife dyad, in which there are two variables from each dyad: behavioral patterns and depression symptoms. This model captures the dependency inherent to couples’ data. In our study, each spouses’ outcome (depression symptoms) is made up of a linear combination of his or her score on the independent variable (actor effect) and his or her spouse’s score on the same independent variable (partner effect). Specifically, an actor effect is the effect of one’s own independent variable on one’s own outcome. For example, the effect of husbands’ behavioral patterns on husbands’ depression symptoms. A partner effect estimates the association between one’s independent variable on one’s partner’s outcome. For example, the effect of wives’ behavioral patterns on husbands’ depression symptoms. Multilevel modeling or structural equation modeling can be used (36). We chose a distinguishable dyad model that differentiates partners in the dyad, based on certain characteristics; in our case, each couple was treated as a distinct dyadic member, based on gender. The significance level for all analyses was set at 5.0% (*P* < 0.05).

## 3. Results

### 3.1. Characteristics of the study population

Participants’ demographic characteristics are shown in Table 1. The mean and standard deviation (SD) age was 25.77 ± 3.35 years for women and 26.83 ± 3.31 years for men who were eligible for the study. The mean (SD) BMI was 22.35 ( ± 4.48) among wives and 23.7 ( ± 3.67) among the husbands. The highest percentage of husbands had college degree or above, at 61.1%, followed by junior high school or below graduates (23.3%), and high school graduates (20.5%). Among wives, 61.1% had college degrees or above, 23.3% had junior high school education or below, and 15.6% had high school education. Compared to wives, husbands had a higher BMI, were less likely to be unemployed in the last month, and earned more per year.

**TABLE 1:** Demographics in childbearing age couples (*n* = 30,559).

Characteristics	Wife (*n* = 30,559)	Husband (*n* = 30,559)	Total (*n* = 61,118)
Age (years), M (SD)	25.77 ± 3.35	26.83 ± 3.31	26.30 ± 3.37
BMI (kg/m^2^), M (SD)	22.35 ± 4.48	23.74 ± 3.67	23.05 ± 4.15
**Education, n (%)**			
Junior high school or below	7,129 (23.33)	7,126 (23.32)	14,255 (23.30)
High school	4,770 (15.61)	6,269 (20.51)	11,039 (18.10)
College degree or above	18,660 (61.06)	17,164 (56.17)	35,824 (58.60)
**Annual Income (yuan/years), n (%)**			
<30,000	8,976 (29.37)	2,166 (7.09)	11,142 (18.20)
30,000–60,000	12,028 (39.36)	7,003 (22.92)	19,031 (31.10)
>60,000	9,555 (31.27)	21,390 (70)	30,945 (50.60)
**Regional areas, n (%)**			
Central Anhui, China	13,547 (44.33)	13,547 (44.33)	27,094 (44.33)
North Anhui, China	7,865 (25.74)	7,865 (25.74)	15,730 (25.74)
South Anhui, China	9,147 (29.93)	9,147 (29.93)	18,294 (29.93)
**Work, n (%)**			
Employment	24,773 (81.07)	29,505 (96.55)	54,278 (88.81)
Unemployment	5,786 (18.93)	1,054 (3.45)	6,840 (11.19)

SD, standard deviation; BMI, body mass index.

### 3.2. Latent classes of health-related behaviors among couples

As shown in Supplementary Table 3, the three-class model was the most parsimonious and substantively sound for husbands and wives. For husbands, the three-class model had lower BIC and AIC than the one- and two-class models and higher entropy values than the four- and five-class models. Similarly, the best fit for wives was achieved using the three-class model.

**FIGURE 2:**
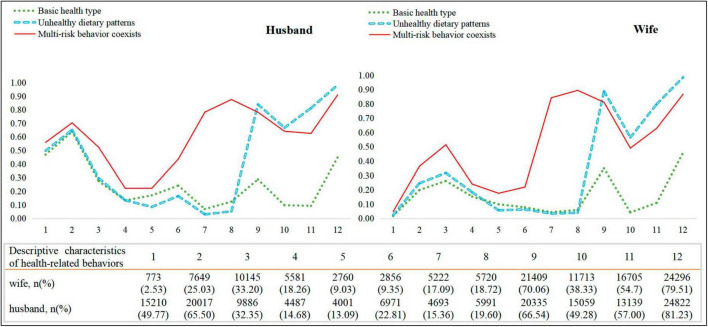
Groups of clustered risk behaviors among husband-wife dyads. 1, Smoking; 2, Drinking; 3, Chronotype; 4, Sitting time; 5, Pickled/fired/barbecued foods; 6, Sugar-sweetened beverages; 7, Takeaway food; 8, Disposable cutlery; 9, Cereals; 10, Fruits and vegetables; 11, Animal-source foods; 12, Legumes and nuts.

Figure 2 presents the item-response probabilities of the three LCA groups from the three-class model. For husbands, the green line labeled the low-risk group named basic health patterns (27.0%), representing the relatively low probabilities of unhealthy behaviors; the blue line labeled the moderate-risk group named unhealthy dietary patterns (58.9%), representing those with the highest possibility of low intake of healthy food, such as legumes and nuts (98.0%), animal protein (81.0%), and cereals (84.0%), but the lowest possibility of other risky behaviors; the red line labeled the high-risk group named multi-risk behavior coexists patterns (14.1%), representing those with the high possibility of smoking (56.0%), drinking (70.0%), and evening chronotype (52.0%).

Among the wives, 30.7% belonged to the low-risk group, 53.0% to the moderate-risk group, and 16.2% to the high-risk group. Specifically, the low-risk group had a relatively positive profile, with low possibility of smoking (2.0%) and low probability of inadequate vegetable and fruit consumption (4.0%). The moderate-risk group had the highest probability of a low intake of legumes and nuts (99.0%), and low intake of cereals (89.0%). The high-risk group showed a high probability of the evening chronotype (51.0%), high intake of takeaway food (84.0%), and high consumption of disposable cutlery (89.0%). For a more detailed probability analysis, refer to Supplementary Table 4.

### 3.3. Correlations between behavioral patterns and depression symptoms in couples

The correlations between the study variables are presented in Table 2. Within-dyad correlations revealed that husbands’ behavioral patterns were significantly related to wives’ behavioral patterns (*r* = 0.15, *P* < 0.01), and husbands’ PHQ-9 scores were positively correlated with wives’ PHQ-9 scores (*r* = 0.12, *P* < 0.01). Husbands’ behavioral patterns were correlated with both their own PHQ-9 scores (*r* = 0.11, *P* < 0.01) and their wives’ PHQ-9 scores (*r* = 0.04, *P* < 0.01). Wives’ behavioral patterns were also correlated with both their own (*r* = 0.12, *P* < 0.01) and their husbands’ PHQ-9 scores (*r* = 0.03, *P* < 0.01).

**TABLE 2:** Correlation between behavioral patterns and depression among childbearing age couples (*n* = 30,559).

	Wives’ behavioral patterns	Husbands’ behavioral patterns	Wives’ PHQ-9 score	Husbands’ PHQ-9 score
Wives’ behavioral patterns	1.00			
Husbands’ behavioral patterns	0.15**	1.00		
Wives’ PHQ-9 score	0.12**	0.04**	1.00	
Husbands’ PHQ-9 score	0.03**	0.11**	0.12**	1.00

**P < 0.01.

### 3.4. Impact of behavioral patterns on depression symptoms at the dyadic level

**FIGURE 3:**
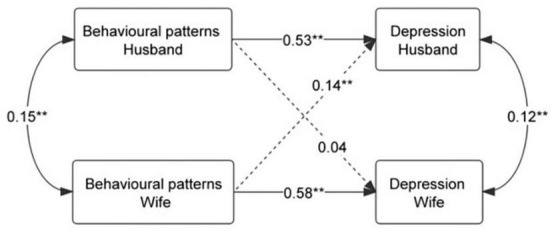
APIM estimates of actor and partner effects of the husband’s and wife’s behavioral patterns on depression. **P < 0.01.

As presented in Figure 3 and Table 3, the APIM analysis indicated that both the husband’s and wife’s behavioral patterns exhibited an actor effect on their own depression symptoms (β = 0.53, *P* < 0.01; β = 0.58, *P* < 0.01). Partner effects differed between genders; only wives’ behavioral patterns had a partner effect on husbands’ depression symptoms (β = 0.14, *P* < 0.01). The partner effect of husbands’ behavioral patterns on wives’ depression symptoms was not significant (β = 0.04, *P* = 0.093). As a supplementary analysis, we examined the effect of each of the health-related behavior variables used in the LCA (Supplementary Table 5). The results were consistent with the main analysis.

**TABLE 3:** APIM estimates of actor and partner effects of the husband’s and wife’s behavioral patterns on depression.

Effect	β (95% *CI*)	
	**Model 1**	**Model 2**
**Actor effect**		
Wives’ behavioral patterns → Wives’ PHQ-9 score	0.57 (0.51, 0.62)**	0.58 (0.52, 0.64)**
Husbands’ behavioral patterns → Husbands’ PHQ-9 score	0.52 (0.46, 0.57)**	0.53 (0.47, 0.58)**
**Partner effect**		
Wives’ behavioral patterns → Husbands’ PHQ-9 score	0.12 (0.06, 0.19)**	0.14 (0.08, 0.20)**
Husbands’ behavioral patterns → Wives’ PHQ-9 score	0.03 (−0.02, 0.08)	0.04 (−0.01, 0.09)

Latent classes of couples’ health-related behavior were included as independent variables, while PHQ-9 score used as the dependent variable. Model 1, unadjusted confounders; Model 2, adjusted for region, age, BMI, education level, and annual income level. **P < 0.01.

## 4. Discussion

Although studies have demonstrated an association between behavioral patterns and depression symptoms, most have focused on a specific health-related behavior, or on individuals (14, 15). The current study uses an LCA model to identify patterns of health-related behaviors. Additionally, the APIM approach was used to assess the relationship between behavioral patterns and depression symptoms among couples of childbearing age. The results showed that both wives’ and husbands’ behavioral patterns had a direct impact on their own depression symptoms. Interestingly, the wife’s behavioral pattern was shown to have an impact on her husband’s depression symptoms, demonstrating a significant partner effect.

Consistent with past research and our first hypothesis, we found that behavioral patterns and depression symptoms were correlated between couples. As couples often have similar or concordant mental and physical health as well as health behaviors, a theory of health concordance has been established (37). Furthermore, a systematic review suggested that both health behaviors and health behavior changes were generally more consistent among couples than among individuals in the general population (38). Even newly-married partners who lived together for a short time shared clear correlations in cardiovascular risk factors and were positively associated with depressive symptoms (39). According to the interpersonal theory of depression, people with depressive symptoms are affected and influenced by their interactions with others (40). Other dyadic studies also support this interdependence. For example, caregiver’s depressive symptoms were significantly associated with care recipient’s (41, 42). In summary, this study highlights the importance of considering the influence of both partners’ behavioral patterns on individuals’ depression symptoms.

Our findings show that wives’ and husbands’ depression symptoms was affected by their own behavioral patterns (actor effect). Previous cross-sectional studies in China have shown that among both male and female participants, those with healthier behavioral patterns reported better mental health status (14, 15). In a prospective cohort study of middle-aged and older adults in Europe, clustered unhealthy lifestyle behaviors were cross-sectionally associated with elevated depressive symptoms, and clustering of two, three, or four unhealthy lifestyle behaviors was prospectively associated with elevated depressive symptoms (43). This finding is consistent with the discussion on the importance of including different lifestyle behaviors in the formulation of policies aimed at preventing mental disorders (44). Each risky behavior may have different mechanisms related to depression. For individuals, the mechanisms by which less healthy behaviors increase the depression risk are not fully understood; however, inflammation may play an important role. For example, diet quality affects immune function and systemic inflammation levels (45), while smoking is associated with increased levels of acute phase proteins (46) and sleep deprivation is associated with alterations in cellular and natural immune functioning (47), thereby inducing depression.

The results of the partner effect analysis were partly consistent with our second hypothesis – that the path between wives’ behavioral patterns and husbands’ depression symptoms was significant, but the path between husbands’ behavioral patterns and wives’ depression was not. If the wife is in the high-risk group, her husband has a significantly higher PHQ-9 score. That is, the depression symptoms of the husband is increased by the poor health behavior of the wife. Similar to our study, Miller and Mason conducted a study that also took into account the dyadic nature of depression. They examined the relationship between marital satisfaction and depression symptoms among 391 married couples, finding that wives’ marital satisfaction had a significantly impact on their husbands’ depression symptoms (48). Besides, they confirms that the individual’s depression is not only affected by his own factors but also by his spouse’s factors.

For couples, there are multiple pathways by which one spouse’s behavioral patterns may influence the other’s depression symptoms. One indirect pathway may be that an individual’s behavioral patterns depend on their partner’s behavioral patterns. Related to this, Umberson argued that many spouses monitor and attempt to control their spouses’ health behaviors (49). This is supported by the theory of interdependence: couple members can transform person-centered motivation into relationship-centered motivation (50). For instance, if one partner maintains a healthy behavioral pattern, both partners may strive to adopt healthy behavioral patterns as goals. One direct pathway is that one spouse’s behavioral patterns may directly lead to changes in another spouse’s depression. In a close relationship, individuals would perceive their partner’s resources, perspectives, and characteristics as part of their own (51). If an individual perceives his/her partner’s behavior patterns as deviating from his/her expectations, it can potentially negatively affect his/her emotional state. Evidence on health behavior change for couples shows that partners have a significant influence on individuals’ health, and the concordance of health behaviors in couples increases over time (38). Therefore, when examining the association between behavioral patterns and depression symptoms in couples, it is essential to consider the husband and wife as a whole, and more evidence should be provided.

One major strength of this study is that we used a large population-based sample of couples to examine the interactions within couples in terms of the relationships between behavioral patterns and depression symptoms. Another advantage is that we identified three distinct behavioral patterns for both wives and husbands, using LCA rather than individual behaviors. This is more in line with reality, as individuals are simultaneously exposed to multiple risky behaviors. Using APIM, we accounted for the interdependence that naturally exists between partners, which is rarely done in depression symptoms research (52). Our findings may hold implications for couple-based interventions, with the goal of reducing the future incidence of depression. Specifically, if a clinician identifies a person with unhealthy behaviors, it may be appropriate to recommend that his/her partner also be evaluated. It will encourage the couple to enhance mutual understanding, engage in joint planning and problem-solving for enacting behavior change, and engage in behavior change as a dyad. A partner can be seen as a coach to help partners change their unhealthy behaviors, as suggested by Baucom et al. (53).

Despite these strong findings, this study has some limitations. First, we used cross-sectional data; therefore, we could not rule out reverse causality. The association between behavioral patterns and depression symptoms is probably bidirectional (54). Second, the duration of cohabitation, which is an important confounding factor (55), was not specifically ascertained among our participants. Future research should consider the duration of cohabitation when estimating the association between behavioral patterns and depression. Third, although we adjusted for as many potential confounders as possible, there are still residual confounders (i.e., mental and physical health, time spent outdoors, and screen time) (44, 56). Fourth, we used the PHQ-9 to assess depression symptom. Although this self-report scale assessment method is very suitable for epidemiological investigation, it will inevitably lead to report bias and misclassification. In the future, prospective studies should elucidate the nature of the causal relationship between behavioral patterns and depression symptoms, and more unhealthy lifestyle factors should be included to confirm the current findings.

## 5. Conclusion

This study adds to the growing body of literature by providing evidence that spouses’ behavioral patterns and depression symptoms are intertwined, which has potential clinical implications. Our findings suggest that couple-based interventions are required to improve husbands’ psychological wellbeing.

## Data availability statement

The raw data supporting the conclusions of this article will be made available by the authors, without undue reservation.

## Ethics statement

The studies involving humans were approved by the Ethics Committee of Anhui Medical University. The studies were conducted in accordance with the local legislation and institutional requirements. The participants provided their written informed consent to participate in this study.

## Author contributions

QY: writing-original draft, formal analysis, and writing-review and editing. XG, ML, and GP: statistical analysis and editing. HG, BW, and YT: manuscript preparation and drafting the manuscript. PZ and SB: writing–review and editing. SS: critical revision and writing-review and editing. FT: conceptualization, funding acquisition, and writing-review and editing. All authors contributed to and approved the final manuscript.
