# Supplementary material for: Association between behavioral patterns and depression symptoms: dyadic interaction between couples
Source: Front Psychiatry. 2023 Nov 16;14:1242611. doi: 10.3389/fpsyt.2023.1242611 (PMC10687217; doi:10.3389/fpsyt.2023.1242611)
Supplement: Supplementary file 1 [file Data_Sheet_1.PDF]

Association between behavioral patterns and depression symptoms: dyadic interaction between couples

Figures S1 Captions

Figure S1. Flow diagram of participants (*n* = 30 559) for this analysis from RHCC, a cohort recruited from China, Anhui area in 2019-2021.

Tables (S1-S4) Captions

Table S1. The assignment of health-related behaviors information results

Table S2. Descriptive statistics of health-related behaviors and depression of childbearing age couples (*n* = 30 559)

Table S3. The fitting index of latent category model for different categories

Table S4. Latent classes of health-related behaviors of childbearing age couples (*n* = 30 559)

Table S5. Associations between health-related behaviors and depression symptoms by generalized linear models

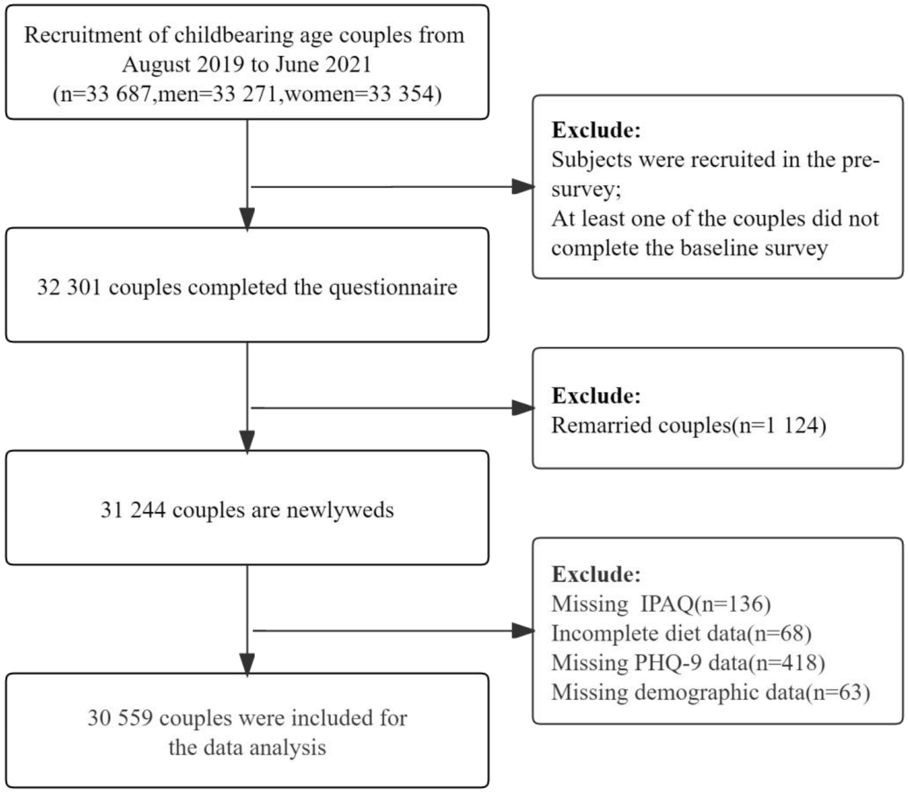

Figure S1. Flow diagram of participants (*n* = 30 559) for this analysis from RHCC, a cohort recruited from China, Anhui area in 2019-2021.

Abbreviations: IPAQ, international physical activity questionnaire; PHQ-9, 9-item Patient Health Questionnaire.

Table S1. The assignment of health-related behaviors information results

Indicators	Assignment
Smoking	yes = smoking, no = non-smoking
Drinking	yes = less than 1 time per week or every week., no = never
Chronotype	yes = late midpoint of sleep, no = early midpoint of sleep
Sitting time	yes = sitting at least 8 hours per day, no = sitting less than 8 hours per day
Pickled/fired/barbecued foods	yes = intake more than three times per week, no = intake less than three times per week
Sugar-sweetened beverages	yes = intake more than three times per week, no = intake less than three times per week
Takeaway food	yes = intake more than three times per week, no = intake less than three times per week
Disposable cutlery	yes = intake more than three times per week, no = intake less than three times per week
Cereals	yes = daily intake and more, no = less than daily intake
Fruits and vegetables	yes = daily intake and more, no = less than daily intake
Animal-source foods	yes = daily intake and more, no = less than daily intake
Legumes and nuts	yes = daily intake and more, no = less than daily intake

Table S2. Descriptive characteristics of health-related behaviors and depression of childbearing age couples (*n* = 30 559)

Health-related behaviors	Wife (*n* = 30 559)	Husband (*n* = 30 559)	Total (*n* = 61 118)
Smoking, *n* (%)			
Yes	773 (2.53)	15210 (49.77)	15983 (26.20)
No	29786 (97.47)	15349 (50.23)	45135 (73.80)
Drinking, *n* (%)			
Yes	7649 (25.03)	20017 (65.50)	27666 (45.30)
No	22910 (74.97)	10542 (34.50)	33452 (54.70)
Chronotype, *n* (%)			
Yes	10145 (33.20)	9886 (32.35)	20031 (32.80)
No	20414 (66.80)	20673 (67.65)	41087 (67.20)
Sitting time, *n* (%)			
Yes	5581 (18.26)	4487 (14.68)	10068 (16.50)
No	24978 (81.74)	26072 (85.32)	51050 (83.50)
Pickled/fired/barbecued foods, *n* (%)			
Yes	2760 (9.03)	4001 (13.09)	6761 (11.10)
No	27799 (90.97)	26558 (86.91)	54357 (88.90)
Sugar-sweetened beverages, *n* (%)			
Yes	2856 (9.35)	6971 (22.81)	9827 (16.10)
No	27703 (90.65)	23588 (77.19)	51291 (83.90)
Takeaway food, *n* (%)			
Yes	5222 (17.09)	4693 (15.36)	9915 (16.20)
No	25337 (82.91)	25866 (84.64)	51203 (83.80)
Disposable cutlery, *n* (%)			
Yes	5720 (18.72)	5991 (19.60)	11711 (19.20)
No	24839 (81.28)	24568 (80.40)	49407 (80.80)
Cereals, *n* (%)			
Yes	21409 (70.06)	20335 (66.54)	41744 (68.30)
No	9150 (29.94)	10224 (33.46)	19374 (31.70)
Fruits and vegetables, *n* (%)			
Yes	11713 (38.33)	15059 (49.28)	26772 (43.80)
No	18846 (61.67)	15500 (50.72)	34346 (56.20)
Animal-source foods, *n* (%)			
Yes	16705 (54.7)	13139 (57.00)	34125 (55.80)
No	13854 (45.34)	13139 (43.00)	26993 (44.20)
Legumes and nuts, *n* (%)			
Yes	24296 (79.51)	24822 (81.23)	49118 (80.40)
No	6263 (20.49)	5737 (18.77)	12000 (19.60)
PHQ-9 score, mean ± SD	3.08±3.51	2.29±3.02	2.69±3.30

Table S3. The fitting index of latent category model for different categories

Class	K	AIC	BIC	aBIC	Entropy	Category Probability
Wife						
Class 1	12	355529.85	355629.78	355591.64	1.00	
Class 2	25	336125.98	336334.17	336254.72	0.69**	0.58/0.42
**Class 3**	**38**	**325315.44**	**325631.88**	**325511.12**	**0.77****	**0.16/0.31/0.53**
Class 4	51	323082.18	323506.88	323344.80	0.77**	0.51/0.12/0.29/0.08
Class 5	64	321249.92	321782.87	321579.48	0.73**	0.13/0.17/0.07/0.36/0.27
Husband						
Class 1	12	411321.68	411421.61	411383.47	1.00	
Class 2	25	392819.39	393027.57	392948.12	0.71**	0.65/0.35
**Class 3**	**38**	**383891.99**	**384208.43**	**384087.67**	**0.77****	**0.14/0.27/0.59**
Class 4	51	381752.01	382176.70	382014.63	0.70**	0.14/0.37/0.31/0.18
Class 5	64	380218.68	380751.64	380548.24	0.71**	0.12/0.35/0.14/0.06/0.32

Note. **BLRT, $P < 0.01$

Table S4. Latent classes of health-related behaviors of childbearing age couples (*n* = 30 559)

Health-related behavior	Group 1: Basic health type (Low risk)		Group 2: Unhealthy dietary patterns (Moderate risk)		Group 3: Multi-risk behavior coexists (High risk)	
	n_H = 8256, 27.01%	n_W = 9 393, 30.74%	n_H = 17 983, 58.85%	n_W = 16 209, 53.04%	n_H = 4 320, 14.14%	n_W = 4 957, 16.22%
Smoking	0.47	0.02	**0.50**	0.02	**0.56**	0.05
Drinking	**0.64**	0.20	**0.65**	0.25	**0.70**	0.36
Chronotype	0.28	0.26	0.30	0.32	**0.52**	**0.51**
Sitting time	0.13	0.15	0.13	0.18	0.22	0.24
Pickled/fired/barbecued foods	0.17	0.10	0.09	0.06	0.22	0.18
Sugar-sweetened beverages	0.24	0.08	0.17	0.06	0.44	0.22
Takeaway food	0.07	0.04	0.03	0.03	**0.78**	**0.84**
Disposable cutlery	0.12	0.06	0.05	0.04	**0.88**	**0.89**
Cereals	0.29	0.35	**0.84**	**0.89**	**0.78**	**0.81**
Fruits and vegetables	0.10	0.04	**0.67**	**0.57**	**0.64**	0.49
Animal-source foods	0.09	0.11	**0.81**	**0.80**	**0.63**	**0.63**
Legumes and nuts	0.45	0.46	**0.98**	**0.99**	**0.91**	**0.87**

Abbreviations: H, husband; W, wife.

Table S5. Associations between health-related behaviors and depression symptoms by generalized linear models

Health-related behaviors[a]	Husband→Husband	Husband→Wife	Wife→Wife	Wife→Husband
	β (95% *CI*)		**β** (95% *CI*)	
Smoking	0.26(0.19, 0.33)	0.30(0.22, 0.38)	1.38(1.13, 1.63)	0.49(0.27, 0.71)
Drinking	0.26(0.19, 0.33)	**0.08(0.00, 0.17)**	0.56(0.47, 0.65)	0.23(0.15, 0.31)
Chronotype	0.35(0.27, 0.42)	**0.00(-0.08, 0.09)**	0.25(0.17, 0.34)	**0.02(-0.06, 0.09)**
Sitting time	0.59(0.49, 0.68)	**0.10(-0.01, 0.21)**	0.72(0.62, 0.82)	0.20(0.11, 0.29)
Pickled/fired/barbecued foods	0.31(0.21, 0.41)	0.18(0.06, 0.3)	0.29(0.15, 0.43)	**0.09(-0.03, 0.22)**
Sugar-sweetened beverages	0.49(0.40, 0.57)	0.17(0.07, 0.27)	0.32(0.17, 0.46)	**0.11(-0.02, 0.23)**
Takeaway food	**0.06(-0.06, 0.17)**	**-0.04(0.17, 0.10)**	**0.08(-0.06, 0.22)**	**-0.01(-0.13, 0.11)**
Disposable cutlery	0.7(0.59, 0.8)	0.32(0.19, 0.44)	0.69(0.56, 0.83)	0.16(0.04, 0.28)
Cereals	0.18(0.10, 0.26)	**-0.09(0.19, 0.00)**	0.24(0.14, 0.33)	**0.06(-0.02, 0.15)**
Fruits and vegetables	0.29(0.22, 0.37)	**0.03(-0.06, 0.13)**	**0.05(-0.04, 0.14)**	**0.03(-0.05, 0.11)**
Animal-source foods	**-0.08(-0.06, 0)**	**0.01(-0.08, 0.10)**	**0.08(-0.01, 0.17)**	**0.04(-0.04, 0.12)**
Legumes and nuts	0.15(0.06, 0.25)	0.33(0.21, 0.44)	0.14(0.03, 0.25)	**-0.01(-0.11, 0.08)**

Note. Health-related behaviors[a]:The control group was "NO" health-related behaviors; *P* value more than or equal to 0.05 is in bold.
